# Detection of Two High Pathogenicity Avian Influenza Virus (HPAIV) Subtypes, H5N1 and H5N5, in a Mass Mortality Event in Wild Seabirds and Co‐Location With Dead Seals

**DOI:** 10.1155/tbed/4680980

**Published:** 2026-01-24

**Authors:** Marco Falchieri, Eleanor Bentley, Holly A. Coombes, Benjamin C. Mollett, Jacob Terrey, Samantha Holland, Edward Stubbings, Natalie Mcginn, Jayne Cooper, Samira Ahmad, Jonathan Lewis, Ben Clifton, Nick Collinson, James Aegerter, Divya Venkatesh, Debbie J. F. Russell, Joe James, Scott M. Reid, Ashley C. Banyard

**Affiliations:** ^1^ Department of Virology, Animal and Plant Health Agency (APHA-Weybridge), Woodham Lane, Addlestone, KT15 3NB, Surrey, UK, vla.gov.uk; ^2^ Surveillance Intelligence Unit, Animal and Plant Health Agency (APHA-Weybridge), Woodham Lane, Addlestone, KT15 3NB, Surrey, UK, vla.gov.uk; ^3^ National Trust, Heelis Kemble Drive, Swindon, SN2 2NA, Wiltshire, UK; ^4^ National Wildlife Management Centre, Animal and Plant Health Agency (APHA), York, YO41 1LZ, UK, vla.gov.uk; ^5^ Department of Biology, University of Oxford, Oxford, OX1 3SY, UK, ox.ac.uk; ^6^ Sea Mammal Research Unit, School of Biology, University of St. Andrews, St. Andrews, KY16 8LB, UK, st-andrews.ac.uk; ^7^ WOAH/FAO International Reference Laboratory for Avian Influenza, Animal and Plant Health Agency (APHA-Weybridge), Woodham Lane, Addlestone, KT15 3NB, Surrey, UK, vla.gov.uk

**Keywords:** avian influenza, Grey Seals, H5N1, H5N5, high pathogenicity, seabirds, zoonotic assessment

## Abstract

H5Nx Clade 2.3.4.4b high pathogenicity avian influenza viruses (HPAIVs) have been detected repeatedly in Great Britain (GB) since autumn 2020, with H5N1 dominating detections but with low level detection of H5N5 during 2025. Globally, these viruses have caused mass mortalities in captive and wild avian and mammalian populations, including terrestrial and marine mammals. H5N1 has been the dominant subtype, and whilst detections have overlapped temporally, occurrences have often been spatially distinct. Here, we report the detection of a mortality event in wild birds on the Norfolk coastline in the East of England, where H5N1 HPAIV was detected in five Great Black‐backed Gulls (GBBGs; *Larus marinus*) and a Northern Fulmar (*Fulmarus glacialis*). Interestingly, at the same site, and as part of the same mortality event, a total of 17 GBBGs, one Herring Gull (*Larus argentatus*), one Atlantic Puffin (*Fratercula arctica*) and one Northern Fulmar tested positive for H5N5 HPAIV. Additionally, H5N5 was also detected in 17 co‐located Grey Seal carcases (*Halichoerus grypus*). The H5N1 HPAIV from an infected bird belonged to genotype DI.2, closely related to contemporaneous detections in GB wild birds and poultry. In contrast, all H5N5 HPAIVs from birds and seals were Genotype I with a 22‐amino acid stalk deletion in neuraminidase (NA) and the 627K polymorphism in PB2. This represents the first recorded instance in GB of two subtypes being detected within the same avian population at the same location. It is also the first mass detection of HPAIV H5N5 in mammals within GB. Potential infection mechanisms are discussed.

## 1. Introduction

H5Nx Clade 2.3.4.4b high pathogenicity avian influenza viruses (HPAIVs) have been consistently detected in Great Britain (GB) since autumn 2020, affecting both avian and mammalian wildlife species and poultry farms [1, 2]. The early phase of the outbreak, from autumn 2020 to spring 2021, was dominated by the H5N8 HPAIV subtype, while H5N1 HPAIV has been the dominant viral subtype detected since summer 2021 [3, 4]. The dominance of the H5N1 subtype has been seen globally with mass mortality events in wild and captive birds and sporadic spillover events into wild, free‐living mammals [2, 5]. Additionally, H5N5 HPAIV has been intermittently detected since autumn 2023 in GB, primarily affecting avian species as well as being sporadically detected across Europe [6], North America [7] and Asia [8]. In Europe, detection in wild birds is often found with marine and coastal species. Interestingly, from an avian perspective, whilst the finding of the H5N1 and H5N5 HPAIV subtypes has overlapped temporally, their detections have typically been epidemiologically distinct.

The detection of mass mortality events across multiple farmed and wild mammals is a recent feature of outbreaks with a virus that has previously affected predominantly avian species, but the scale of the epizootic in reservoir species seems to be the main cause of these spillover events where distribution and ecologies overlap. This is particularly true of outbreaks in marine mammals, such as those seen in South America, where a range of pinniped species, including carnivorous aquatic mammals such as seals, have been involved [9–11] and genetic assessment of viruses has demonstrated the interplay of the virus between avian and mammalian hosts. This has also been demonstrated in sub‐Antarctic environments, where mortality in birds has led to mortalities in Antarctic Fur Seals (*Arctocephalus gazella*) and Southern Elephant Seals (*Mirounga leonina*) [12, 13]. A further dimension has been observed in farmed mammalian species including mink [14], foxes [15], sheep [16] and most notably dairy cattle (in the United States of America) [17]. The latter has increased the zoonotic potential of this virus and pandemic risks through the excretion of very high titres of virus in milk from infected dairy cattle [18–20]. Not only has this led to increased risk of human exposures to the virus, albeit generally with mild infection outcomes, but both wild and domestic avian and mammalian species have also succumbed, likely through exposure to infected milk [17]. A further risk to animal health has come through environments where captive (e.g., zoological collections) [21] or domesticated animals (e.g., pet cats) have been fed contaminated avian material as foodstuffs [22]. Although comparatively rare, this route of exposure has been reported on numerous occasions with significant outcomes as animals have succumbed to infection via this route. From the GB perspective, in wild free‐living mammals, sporadic detections of infection with HPAIV H5Nx have been detected [23], but no mass mortality events have been described.

Influenza A virus infections of seals are poorly understood and are only sporadically detected, with other pathogens generally being responsible for mortality events. There are two species of seals that live in the British Isles, Grey Seal (*Halichoerus grypus*) and Common Seal or Harbour Seal (*Phoca vitulina*), but seal populations vary in size and location. One key area where seal populations visit and are monitored in GB is Blakeney Point (52.9752°N, 0.9921°E), Norfolk [24]. Around 10% (>7400 pups) of GB Grey Seal pups are born at Blakeney Point, and it is the largest seal breeding colony in England [25]. The GB breeding figures account for approximately 35% of the global and approximately 90% of the North‐East Atlantic populations [25]. During the breeding season at Blakeney between November and January, females congregate to give birth to a single pup which they suckle for approximately 18 days before pups moult their white coats and take to the sea at approximately 31.5 days. The females come into oestrus at weaning, attracting the presence of males. The breeding season is asynchronous so not all seals will be at the same location at the same time,but, nevertheless, approximately 20,000 Grey Seals, including males, females and pups, likely visit Blakeney Point during the breeding season [24]. This substantial and dense aggregation suggests the potential for two‐way transmission of disease between seals and birds, although mainly gull species, with likely exposure to seabird faeces and carcases on the shoreline and seal haul out, and the scavenging by birds of seal afterbirths and dead pups by gulls [26, 27]. There is likely exposure to seabird faeces, as well as to dead birds, in the pupping areas where large gulls, such as Herring Gulls (*Larus argentatus*) and Great Black‐backed Gulls (GBBGs; *Laurus marinus*), feed on dead seals and afterbirths. Other birds such as the Puffin and Fulmars had likely been washed in by the sea, but infected dead bodies could still provide an interface for pathogen exchange.

Here, we report the occurrence of a mass mortality event in wild birds in the East of England, where both H5N5 and H5N1 HPAIV were detected in gulls alongside concomitant infection of Grey Seals with H5N5. This represents the first recorded instance in the United Kingdom of H5N1 and H5N5 being detected as co‐circulating at the same location and within the same species, as well as the first detection of HPAIV H5N5 in mammals. We overview the case history, clinical manifestations and epidemiological investigation, along with virological, molecular and bioinformatic analyses that confirm these findings and hypothesise infection routes and the potential directionality of infection based on details of the incident.

## 2. Materials and Methods

### 2.1. Clinical and Epidemiological Investigation

An avian mortality event investigation was triggered on 5 February 2025, following a report to the Department for Environment, Food & Rural Affairs (DEFRA) online reporting system [28] of bird carcases on National Trust land at Blakeney Point, Norfolk. This is a relatively remote extensive dune and sandy beach coast, which is managed as a nature reserve and is protected by numerous official conservation designations. The foreshore is wide and long, with numerous sandbars on which seals haul out and breed. Samples from bird carcases were taken in line with government guidelines to determine wild bird species affected, their number, the level of environmental contamination, as well as the distribution of viral subtypes involved. Seal carcases were also observed during the preliminary field visit, at a time where a natural baseline of approximately 5% mortality was expected [29]. Considering recently reported HPAIV H5Nx cases in pinnipeds across the globe and the potential for mass mortality events potentially including mammal‐to‐mammal transmission, a further epidemiological investigation was carried out to assess whether any of the dead seal mortalities were positive for influenza virus as well as to determine the range of species affected, the level of environmental contamination and the distribution of HPAIV subtypes involved.

Multiple samples were collected from a range of avian and mammalian carcases as well as avian faecal material (*n* = 8), feathers (*n* = 3) and a water sample (*n* = 1) as part of the environmental and virological assessment. Samples from carcases included swabs from orifices (oral, nasal and rectal for mammals, and oropharyngeal [OP] and cloacal [C] for birds) and samples from target internal organs, either swabs or tissues, such as brain, lung and miscellaneous. These tissues were selected as key site for virus detection in these species [12].

### 2.2. Virological Investigation

All samples were processed within Specified Animal Pathogen Order (SAPO) Level 4/Advisory Committee on Dangerous Pathogens (ACDP) Level 3 compliant high containment facilities at the Animal and Plant Health Agency. Sample processing depended on sample type and matrix and was processed as described previously [30]. Briefly, tissue samples were homogenised for extraction whilst swabs samples were processed by cutting the swab tip into 1 mL serum‐free Leibovitz’s L‐15 medium (Gibco) containing antibiotics (penicillin and streptomycin), incubated at room temperature for 10 min before standard viral RNA (vRNA) extraction. Brain and lung tissues were prepared as a 10% (w/v) suspension in L‐15 medium and incubated at room temperature for 60 min before using standard RNA extraction protocols [31]. RNA was extracted using the MagMAX CORE Nucleic Acid Purification Kit (ThermoFisher Scientific, United Kingdom) as previously described [31]. Extracted RNA was assessed for vRNA using RT‐PCR assays specific for M gene [32], an H5 HPAIV [33] and/or neuraminidase (NA) [34]. A standard curve was generated using a 10‐fold dilution series of titrated H5N1 HPAIV RNA as previously described to assess test efficiency [31].

### 2.3. Genomic Analysis

Whole genome sequences (WGSs) were generated from positive samples using Oxford Nanopore Technology as described previously [12]. Briefly, extracted vRNA was converted to double‐stranded cDNA and amplified using a one‐step RT‐PCR using SuperScript III One‐Step RT‐PCR Kit (Thermo Fisher Scientific), see Supporting Information 1: Table S1 for specific primers. PCR products were purified with Agencourt AMPure XP beads (Beckman Coulter) prior to sequencing library preparation using the Native Barcoding Kit (Oxford Nanopore Technologies) and sequenced using a GridION Mk1 (Oxford Nanopore Technologies), according to manufacturer’s instructions.

Assembly of the influenza A viral genomes was undertaken using a custom in‐house pipeline previously described [4]. Comparison of the study‐derived sequences and contemporary H5 sequences was undertaken against all avian H5 sequences available on GISAID between 1 January 2020 and 28 February 2025. All sequences were aligned on a per segment basis using MAFFT v7.520 [35] and trimmed against a reference using SeqKit v2.5.1 [36]. The trimmed alignments were used to infer maximum‐likelihood phylogenetic trees using IQ‐TREE Version 2.2.5 [37] along with ModelFinder [38] and 1000 ultrafast bootstraps [39]. Ancestral sequence reconstruction and inference of molecular‐clock phylogenies were undertaken using TreeTime v0.10.1 [40]. Phylogenetic trees were visualised using R Version 4.3.3 with ggplot2 Version 3.5.1 [41] and ggtree Version 3.14.0 [42]. Sequences were genotyped from phylogenetic trees by comparison to known reference sequences for all genotypes currently circulating in GB. Sequences derived from both seals and avian species were assessed for the presence of adaptive mutations that may confer increased replication in mammals. All sequences were aligned on a per segment basis using MAFFT v7.520 [35] and manually trimmed to the open reading frame using Aliview Version 1.28 [43]. Trimmed sequences were translated to amino acids and visually inspected for mutations. All influenza sequences generated in this study are available through the GISAID EpiFlu Database (https://www.gisaid.org, Supporting Information 1: Table S2).

## 3. Results

### 3.1. Clinical and Epidemiological Investigation

**Figure 1:**
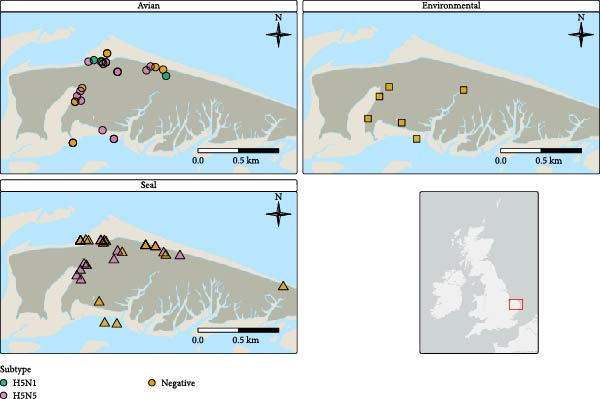
Spatial representation of samples collected from birds, the environment and seals and H5N1 or H5N5 HPAIV testing results.

An investigation into a mortality event involving gulls and other avian species was initiated at Blakeney Point, Norfolk (Figure 1), on 5 February 2025 under the avian influenza wild bird surveillance scheme [28]. Carcases were in varied states of decomposition and often incomplete, sometimes discovered as disarticulated limbs, heads and so forth. In addition, the beach was extensive and a complete search in the time available between tides was impossible. As such, a precise carcase count could not be defined, but the likely number of dead birds far exceeded the number sampled. In total, five birds including four GBBGs (*Laurus marinus*) and one Herring Gull (*Larus argentatus*) were identified in the field and sampled; four Grey Seal carcases were also sampled (Table 1 and Supporing Information 1: Table S3).

**Table 1:** Summary of animals sampled and HPAIV H5N1 and H5N5 positive animals during the incident.

Species	First sampling (5 February 2025)			Second sampling (14 February 2025)		
	H5N1	H5N5	Total sampled	H5N1	H5N5	Total sampled
GBBG	2 (50.0%)	1 (25.0%)	4	3 (14.3%)	16 (76.2%)	21
Herring Gull	—	—	1	—	1 (50.0%)	2
Atlantic Puffin	—	—	—	—	1 (100%)	1
Northern Fulmar	—	—	—	1 (50.0%)	1 (50.0%)	2
Razorbill	—	—	—	—	—	1
Grey Seal	—	2 (50.0%)	4	—	15 (37.5%)	40
Total	2	3	9	4	34	79

Abbreviation: GBBG, Great Black‐backed Gull.

The results from these samples prompted a secondary sampling undertaken on 14 February 2025, whereby a total of 40 Grey Seal carcases were sampled as well as 27 avian carcases of various species including 21 GBBGs, two Herring Gulls, one Atlantic Puffin (*Fratercula arctica*), two Northern Fulmars (*Fulmarus glacialis*) and a Razorbill (*Alca torda*) (Table 1 and Supporing Information 1: Table S3).

Mortality counts carried out by on‐site staff at the end of January and beginning of February 2025 showed that an unusual die‐off had taken place in Laridae with 46 GBBGs, both juveniles and adults, plus one Herring Gull and one Common Gull (*Larus canus*) being noted. However, the exact timing of the die‐off is undefined as mortality counts are only performed at the end of the seal breeding season with timelines spanning from November 2024 up to February 2025. Most avian carcases showed degrees of decomposition, mummification and scavenging, indicating that the avian die off had probably been ongoing throughout the winter. Blakeney Point is home to England’s largest Grey Seal colony [24]. The site hosts and is visited by numerous wild birds including Anatidae such as ducks, geese, swans and Laridae species. No large mortality events were recorded in seal pups during the 2024–2025 breeding season. Seal carcase counts undertaken between 31 January 2025 and 3 February reported 177 seal carcases present on site with only eight carcases being adults and the remaining 169 carcases constituting pups, weaners or immature seals. These mortality levels were within an expected mortality range for the colony during the breeding season. Further surveys, conducted after 3 February, estimated a total of up to 15 dead adults and 200 dead pups, including weaners and immatures.

### 3.2. Virological and Environmental Investigations

Upon initial sampling 50% (*n* = 2/4) Grey Seals and 60% of avian sampled carcases (*n* = 3/5) tested positive for HPAIV, the latter including two GBBGs infected with HP H5N1 and one GBBG positive for HPAIV H5N5 (Table 1 and Figure 1). Following a second sampling effort, 37.5% (*n* = 15/40) Grey Seals tested positive by PCR for HPAIV H5N5; no pinnipeds were positive for H5N1 HPAI. From an avian perspective, for the second sampling, a total of 27 birds were sampled: 76.2% (*n* = 16/21) GBBGs, 50% (*n* = 1/2) Herring Gull, 100% (*n* = 1) Atlantic Puffin and 50% (*n* = 1/2) Northern Fulmars tested positive for H5N5 HPAI; 14.3% (*n* = 3/21) GBBGs and 50% (*n* = 1/2) Northern Fulmar tested positive for H5N1 HPAI (Table 1 and Figure 1). Results per bird and sample type are shown in Supporing Information 1: Table S3.

### 3.3. Genomic Analysis

Eleven H5N5 HPAIV WGSs were recovered from a total of 16 H5N5 clinical samples that were sent for sequencing (Supporting Information 1: Table S2). Six WGSs were recovered from seals and one Herring Gull, one GBBG and one fulmar, with all seal‐ and avian‐derived viral sequencing being H5N5 Genotype I according to the EURL classification (Supporing Information 2: Figure S1). Mutational analysis demonstrated the presence of a 22‐amino acid NA stalk deletion and a PB2 E627K polymorphism in all H5N5 samples.

One WGS was recovered from a GBBG, from a total of four H5N1 clinical samples that were sent for sequencing. This WGS belonged to the DI.2 genotype, which has been the most prevalent in wild birds in the United Kingdom during winter 2024–2025. No mutations suggestive increased mammalian adaptation or increased zoonotic potential were found in the H5N1 sequence.

## 4. Discussion

Incursions of HPAIV Clade 2.3.4.4b H5Nx viruses into GB and the Europe through migratory bird movements have occurred repeatedly since October 2020. The high‐risk season for HPAIV incursion in GB typically starts each October coinciding with the beginning of the arrival of migratory wildfowl. This period also sees distinct behavioural changes in resident wildfowl which begin to aggregate on their overwintering grounds, often the same locations used by overwintering migrants. Occasional spillover events into commercial poultry and captive bird collections can result in severe outbreaks of notifiable disease. Such outbreaks are controlled through rapid reporting, diagnostic testing and movement restrictions, with culling of affected flocks to prevent further spread. As well as wild bird activity leading to infection of poultry, the reinfection of wild bird species from infected poultry has also been identified [44], although the frequency of bidirectional transmission is poorly understood. Significant mortality has also been seen in populations of wild birds [45–47], occasionally affecting species of conservation concern. High levels of HPAIV‐driven wild bird mortality increase infection pressure for mammalian species, especially scavengers.

The first mammalian wildlife detections of H5Nx in GB occurred in captive Common Seals, Grey Seals and a Red Fox (*Vulpes vulpes*) at a wildlife rehabilitation centre [48]. Diagnostic investigations linked the infections to a mortality event in Mute Swans (*Cygnus olor*) at the centre in the weeks preceding the detection in mammals. This initial detection involved the Clade 2.3.4.4b H5N8 subtype, and mammalian adaptations linked to increased zoonotic risk within viral sequences were limited to E627K and D701N within the PB2 gene [48]. Since 2021, Clade 2.3.4.4b H5N1 has emerged as the dominant subtype across GB, Europe and the rest of the world with a vast array of genotypes being detected as part of that clade of H5Nx viruses [4, 49]. More recently, global detections of infection in different mammalian carnivore species [3, 4, 9–15] as well as herbivore/omnivore species [16, 17, 50, 51] have further underlined the ability of these viruses to cross the species barrier. Most recently, in GB, detection of H5N1 HPAIV (genotype DI.2) was confirmed in a single sheep, demonstrating risk across different sectors where infection pressure is high [16].

Although the initial Clade 2.3.4.4b epizootic was caused by the H5N8 subtype, it was subsequently displaced by H5N1, which went on to dominate the global avian influenza landscape. Alongside, a further subtype, H5N5, has also been detected in different environmental contexts [7, 8]. In GB, HP H5N5 (Genotype I) was first detected in autumn 2023 with detections mainly in birds of prey and gull species. H5N5 was detected in 131 found dead wild birds and one poultry premise between October 2024 and 28 February 2025 (through the statutory surveillance in wild birds) [52]. Until the event described here, H5N5 had never been detected in non‐avian species in the British Isles.

The detection of H5N5 in both seals and gulls at Blakeney Point raises several questions regarding the infection and transmission kinetics of these viruses. The initial reason for sampling was a mortality event in gulls and in the weeks preceding this event, HP H5N1 wild bird positives were detected in the area predominantly associated with infection of Anatidae, birds of prey and gulls. All of the earlier detections were of the H5N1 DI.2 genotype that had become dominant both within the EU and across GB [53]. No local detections of H5N5 had been made until this event was investigated. It is not clear why a few gulls tested positive for H5N1 whilst most gulls and other avian species were positive for H5N5. Further, the dynamics of infection both to and between the seals is unclear. Sampling of the seals was reactive to the avian die‐off and so could have been missed as the mortality levels seen were not enough to trigger concern for seals at this heavily populated breeding site and as such did not indicate a risk for HPAIV infection in seals. Environmental samples all tested negative suggesting that environmental contamination was low although in a dynamic and aggressive littoral coastal environment, both carcases and environmentally relevant material (e.g., faecal material) may have been lost.

Recent studies have suggested that mammal‐to‐mammal transmission has occurred with H5N1 genotype B3.2 viruses in South America, where significant mortality events have been seen [9–13]. The mortality level at Blakeney Point does not support that there was any mammal‐to‐mammal infection occurring, although the potential for this cannot be ruled out. No further cases of H5N5 in seals were detected in GB or reported from Europe. Grey seals in GB are part of a pan‐European metapopulation and exhibit partial migration [54]. Interestingly, one female seal (cow), which was found dead in this incident, had been tagged at the Ecomare seal centre on the Dutch Wadden Sea Island of Texel on 29 December 2014 as a mother‐dependent whitecoat pup (pers. comm. Seal Centre Ecomare). A higher proportion of the European metapopulation spend the breeding season in the United Kingdom compared to the rest of the year. As such, a substantial proportion of seals that breed at Blakeney Point likely subsequently travelled to continental Europe, particularly the Wadden Sea [55]. Even those remaining in South East England likely moved between haul‐out sites (locations on land where seals come ashore to rest, breed or give birth) [56]. As well as travelling offshore, Grey Seal foraging sites extend up to 450 km from a haul‐out sites [57], and pups typically disperse more widely than adults [58]. Such movements mean that in the advent of mammal‐to‐mammal transmission, infection could spread widely through the North‐East Atlantic Grey Seal population. Moreover, Grey Seals often haul‐out in the vicinity of harbour seals. Indeed, Blakeney Point is part of The Wash & North Norfolk Special Area of Conservation (SAC), designated for Harbour Seals (the other seal species present in GB and of conservation concern). The SAC holds most of the English harbour seal population and has recently undergone unexplained declines. The Grey seals have previously played a role in spreading disease (phocine distemper virus), which has had a huge impact on the less wide‐ranging Harbour Seal [59].

Infection of seals must therefore be likely attributed to close interactions with infected seabirds. These densely packed and dynamic seal colonies attract a regular traffic of wild birds to scavenge moribund and dead seals and in turn seals may scavenge infective wild birds, which succumb to disease or become exposed to HPAIV from infective guano left by foraging birds. Ingestion of seabirds by seals on the breeding colony is considered unlikely, and it is more plausible that infection through interactions such as playing with carcases or inhalation of infectious material during investigative behaviours around bird carcases is responsible, although this cannot be definitively concluded.

A further interesting point is that all H5N5 sequence derived from the seals and gulls contained both the NA stalk deletion and the E627K mutation associated with mammalian adaptation [60], further raising uncertainty on the directionality of infection. The most recently detected H5N5 sequences from different avian species included the stalk deletion but had a 627E residue at that position in PB2 [61]. However, assessment of global H5N5 sequence from avian species across the globe from 2020 onwards has demonstrated that the E627K mutation can be detected in avian species in the absence of obvious mammalian involvement [6, 7, 61]. The ability of birds to maintain viruses with mammalian adaptations is a further area of considerable interest [9–13]. This phenomenon requires further investigation.

The co‐circulation of distinct subtypes and genotypes within species occupying the same temporal, spatial and ecological landscapes raises important questions regarding the potential for viral reassortment and the role of gulls in the emergence of novel genotypes. Numerous H5N1 genotypes have been described in each previous season dominated by H5N1 viruses, although a few genotypes were detected more frequently [4, 53]. Interestingly, since the beginning of the 2024/25 AIV season in GB, genotype diversity has been limited with DI.2 dominating positive avian detections [61]. Alongside DI.2, only the BB genotype, a previously dominant genotype, has been detected with some regularity, predominantly in the South‐West of England in gull species that links with occasional detections in continental Europe [53]. Further, there have been two detections of the DI.1 genotype in wild birds in 2024, both have been of Norfolk, and it has not been detected since. Interestingly, the contemporary H5N5 detections appear to be entirely represented by a single genotype (Genotype I), with no evidence of reassortment. Further, since the emergence of the H5N1 BB genotype in 2022, this has also exhibited predominately in gull species with limited reassortment. How and why different HPAIV genotypes are generated, and either dominate or disappear, remains a significant knowledge gap, although several studies have demonstrated differential shedding and clinical impact in poultry species, and it is likely that similar occurs in wild birds.

In conclusion, the detection of two subtypes, H5N1 and H5N5 in avian species linked to a mammalian mortality event that appears restricted to infection with a single H5N5 subtype, is of high interest. Conditions for sampling carcases were sub‐optimal, and the environmental conditions within which these detections were made precluded further swabbing and assessment. Regardless, it is important to define and describe such detections as they clearly demonstrate that different viral subtypes can be present within a mortality event without reassortment being detected. Understanding the intra and inter‐species infection and transmission dynamics is of high interest.

## Disclosure

The views and opinions expressed are those of the authors only and do not necessarily reflect those of the European Union or REA. Neither the European Union nor the granting authority can be held responsible for them.

## Conflicts of Interest

The authors declare no conflicts of interest.

## Funding

This work was supported by the Department for Environment, Food and Rural Affairs, UK Government (Grants SE2230, SV3032, SV3400, SE2227, and OMO402), Biotechnology and Biological Sciences Research Council (Grant BB/Y007271/1), Natural Environment Research Council (GrantNE/Y006194/1) and UK Research and Innovation fellowship (BB/W009404/1).

## Supporting Information

Additional supporting information can be found online in the Supporting Information section.

## Supporting information


**Supporting Information 1** Table S1: Oligonucleotide primers used for cDNA synthesis and amplification for whole genome sequencing. Table S2: GISAID accession numbers for all sequences generated in this study. Table S3: Samples collected and results.

**Supporting Information 2** Figure S1: Maximum‐likelihood phylogeny of European H5 influenza sequences from GISAID, subset to 0.5% sequence divergence, used to contextualise how HA sequences generated in this study compare to previously released European data. Tip labels are coloured by subtype.

## Data Availability

The data that support the findings of this study are openly available on GISAID at https://gisaid.org/.
