# Supplementary figures and images for: Detection of Two High Pathogenicity Avian Influenza Virus (HPAIV) Subtypes, H5N1 and H5N5, in a Mass Mortality Event in Wild Seabirds and Co‐Location With Dead Seals
Source: Transbound Emerg Dis. 2026 Jan 24;2026:4680980. doi: 10.1155/tbed/4680980 (PMC12831281; doi:10.1155/tbed/4680980)

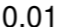

Supplement: Supplementary file 2 — Supporting Information 2 Figure S1: Maximum‐likelihood phylogeny of European H5 influenza sequences from GISAID, subset to 0.5% sequence divergence, used to contextualise how HA sequences generated in this study compare to previously released European data. Tip labels are coloured by subtype. [file TBED-2026-4680980-s001.pdf]
